# Congenital Hydrocephalus

**Published:** 1856-09

**Authors:** Wm. Hales Hingston

**Affiliations:** Montreal


### SELECTIONS.

### *Congenital Hydrocephalus, with remarks.* BY WM. HALES HINGSTON, M.D., L.R.C.S.E., &c.

Hydrocephalus, like accumulations of water in any other part or organ, is a disease of debility, proceeding from a relaxed condition of the secernents of a part; from inactivity of its absorbents, or, as more frequently happens, from both; the *cause* of the disease being rarely manifest.

Accumulations of fluid are met with in various parts within the cranium: "inter integumenta ipsa externa; inter haec et cranium; inter cranium et cerebri membranas; inter membranas ipsas; harumque duplicaturas; inter has et cerebrum; inter cerebri plicas; in cavitatibus ipsis."* The fluid, once secreted in any part, spreads with little resistance to another. If the disease occur in infancy, (and infants are most generally the subjects of it,) the bones of the cranium, not yet united by their bony sutures, yield to the internal pressure. Somewhat later in childhood, and while the *fontanelles* are yet unclosed, they, by a preternatural fullness,† or bulging outwards, warn us of the mischief going on within the skull.

Hydrocephalus is occasionally congenital, sometimes rendering the head so large, as greatly to impede, and add to the danger of, delivery.

The appearances, which these congenital malformations present, are not uniform. In the majority of cases, the whole head enlarges gradually; but, in not a few, we may observe protrusion of one side only; while in a still smaller number, an egg or pear shaped tumor is visible beneath a *fontanelle* or an attenuated parietal or other cranial bone.

"The mode of origin or pathogenesis of congenital hydrocephalus differs most probably in no essential particular from that of the chronic hydrocephalus which commences in the extra uterine periods of life. * * * * The general arrangement of the skull of the fœtus, and the manner in which the cerebrum itself is developed, are both highly favorable to an excessive accumulation of serum. And I believe that the really essential part of congenital hydrocephalus, that which arrests the development of the brain, is the affection of the epididyma; that in proportion to the degree to which the hydrocephalus has advanced, and according to the period of fœtal life at which it commenced, it does, in various manners and to different extent, arrest the development of the brain, and occasion monstrosity of it; and so far contains the ground of its alliance with hemicephalus, hydrencephalocele, singleness of the cerebrum (cyclopia) &c."‡

---

* Van Swieten, "*Commentaria.*"

† I speak generally, and not in ignorance of the fact, that in some cases of hydrocephalus, the fontanelles are depressed.

‡ Rokitansky's Pathological Anatomy, Vol. III, p. 276, Amer. ed.

The substance of the brain in this affection, resembles the *ramollisement* of French pathologists. Sometimes the whole organ, sometimes the portion in immediate contact with the water undergo softening.

So much then, for general remarks, by way of a preface to a case which came under my observation some time ago.

Mrs. W., a stout healthy woman, *æt* 30, sent for me on the morning of the 4th September last. I was told that she was suffering from violent labor pains, which threatened abortion—that a midwife had been in attendance during the whole night, but that no progress had been made. I found the woman on my arrival on her back, with knees in a flexed position, countenance expressive of great suffering, eyes suffused and red, skin hot, pulse 115, hard and wiry.

The midwife* was on her knees in the patient's bed, both hands beneath the counterpane, lips compressed, tugging away during a "pain(?) most energetically, and perspiring as copiously as if the fee would be regulated by the visible amount of cutaneous exhalation. On making a vaginal examination, I found the *labiæ*, from the unwarrantable handling they had received, very much tumified, hot and painful; the *os uteri* not dilated, but tilted forwards behind—I might almost say, *above* the symphisis of the pubis—so high, indeed, that with difficulty could the tip of the finger be brought near its edge. A large tumor—tender on pressure—occupied the hypogastric region. Recognising this as a distended bladder, I introduced the catheter, and drew off fully two quarts of dark offensive urine, with sudden and complete relief. The rectum, also in a loaded state, was emptied by castor oil. During the three following days the catheter required to be used twice daily, and at the end of that period the retroversion was reduced, and the uterus ascended to the superior strait.

I saw nothing more of my patient until two o'clock on the morning of the 13th March. She was then in labor; the *liquor amnii* had escaped the day before. The pains were severe, and at short intervals. On examination, the breech of the child was found to be presenting. During a remission of pain, the feet were brought down, and the body soon followed, but the chin, by a violent pain, was forced against and rested upon the sumphisis, and any attempt at altering its position immediately induced violent pains. Having succeeded, eventually, in placing the head in a more favorable position, every attempt at extraction was made for upwards of two hours, but without avail. At length I resolved upon diminishing the bulk of the child's head—a resolution which cost me but little pain, as the pulsation in the funis had ceased upwards of an hour and a half before. At this stage of the proceedings, I was joined by the professor of midwifery, McGill University, Dr. Hall, who fully coincided with me in the opinion that craniotomy afforded the best possible chance of safety to the mother.

The patient, therefore, having been placed upon her left side, the body of the child was drawn towards the back of the mother by Dr. Hall, (who had already very dexterously placed the head in the "1st position" of Naegele, and who, with myself, had fruitlessly endeavored, by depressing the chin, to accomplish delivery in that way,) forming an

---

* I think it but justice to state that the self-styled midwife is an unqualified and unlicensed woman.

obtuse angle at the neck. Introducing my left index finger, I passed it upwards as far as the obstructed nature of the passage would admit, and, guided by it, introduced the perforator, entering the neck at a point corresponding with the sixth cervical vertebra. Partly by a cutting, partly by a sawing motion, the instrument soon reached the cranial cavity, when, on opening it, a gush of fluid escaped from between the handles; the bones of the skull then collapsed, and the whole slipped easily away. The patient, although in a highly excited state from the consciousness of having such a formidable instrument within her, admitted, during the operation, and afterwards, that she experienced no pain whatever.

On observing the child, we were struck with the enormous size of its head, which, on measurement, was found to be as follows:*

| | |
|---|---|
| From occipital protuberance over vertex to nasal spine, | 22¾ inches. |
| Greatest circumference of head, | 29½ " |
| Altitude, | 6 " |

There was exaggerated strabismus of both eyes; the body was well formed, and of the usual size of a male† child at that period. All the fingers and toes of the body were permanently flexed, and talipes varus of both feet completed the deformity of this truly ugly little specimen of mortality.

On opening the skull from above, the membranes were found to be thickened, and of a deep red color. The pia mater, firmly glued to the arachnoid, was dotted here and there with a pale cretaceous substance, intimately united with it. About a half pint of serous fluid still floated over the cerebral surface of the base of the skull; a film of cerebral matter, about one line in thickness, thickly studded with tubercular matter lined the membranes, except at the superior surface, where it became gradually thinner, and was ultimately lost; so that the fluid must, some weeks prior to birth, have escaped from its cerebral covering, and been converted into a hydrencephalocele. The small mass of matter representing the brain, and resembling softened cortical substance, bore no resemblance, except in color, to what it should have been; no convolutions or irregularities were visible on its surface. The *optic nerves hung loosely in the cavity*, but no trace of others could be detected. The upper part of the spinal cord seemed to have undergone absorption, for no part of it could be detected from that point of view, and friends seemed indisposed to permit an examination from behind.

To a few points of interest in the above case, I would wish briefly to draw attention, in the order in which they have been related, and—

1stly. *Retroversion.* Retroversion of the uterus, in the early months of pregnancy, is the result, generally, of some mechanical force applied to that organ. We may readily understand how easily a preternaturally

* It must be borne in mind that these measurements are proximately, not absolutely, correct. The bones of the skull were, as nearly as possible, filled out to their previous dimensions, but the absence of the contained fluid rendered precision impossible. Moreover, the forcible traction employed, previous to resorting to craniotomy, must have interfered with the shape, perhaps increased the size of the skull.

† Dr. Georget, in the "London Medical Repository," Vol. 14, observes: Monstrous, diseased, or mal-formed fœtuses, are generally of the *female* sex. "Is it," he enquires, "that this sex possesses less energy of organization than the male? or that the generative power is longer and more perfect, in the latter than the former?"

distended bladder may tilt over the fundus, and leave it in the hollow, or resting on the promontory of the sacrum. In this case the uterus was in a position nearly the reverse of natural; the fundus pressing against the rectum, the os behind the symphysis and against the neck of the bladder—preventing, in this way, the action of these two emunctories.

2ndly. *Retention of Urine.* At first the cause, afterwards the result of the displacement of the uterus. In this case, so little inconvenience was felt from the distention of the bladder, that the patient thought I was directing too much attention to it, and was not a little surprised at the relief which followed its evacuation. The pains, moreover, were of a character to mislead; they were strong, "bearing down pains," which the patient aided, by forcibly pulling at a bandage tied to the bed-post, for the expulsion of the fœtus, as she thought—a condition, which, if not speedily relieved, would have occasioned rupture of the bladder.

3dly. *Breech Presentation.* In Denman's midwifery we read the following:—"It is some comfort to women to be informed, and I believe the observation is almost universally true, that affections of this kind (dysuria) are never produced, except in those cases in which the presentation of the child is natural." If Denman's observation be correct, this case must be considered a rare, if not an unique exception; although I can really perceive no reason why exceptions should not be of frequent occurrence.

4thly. *Craniotomy.* Craniotomy in head presentations, is, by obstetricians, considered to be one of the easiest operations which could, for the extraction of the fœtus, be performed. Facility, however, vanishes in presentations of the breech and feet. The head, if large, or even if of average size, with contracted pelvis, lies so high in the "brim," that the obstetrician's finger cannot always afford a safe guide to the point of the instrument. In the case under consideration, the whole head, with the exception of the depressed chin, was entirely above the pectineal line. Had the bones of the skull been lined by the ordinary brain matter, collapse might not have followed perforation, and labor might have required to be terminated in some other way; but, notwithstanding this apparent objection, it appears to me reasonable to attempt evacuation of the head through the passage formed in the long axis of the neck, rather than to thrust an instrument unprotected into the cranium, probably, but possibly between the wall of the vagina and uterus, or into the uterus itself. The additional injury to the child would be of small moment, as the operation would not be undertaken until long after the child had ceased to exist.

"Doctors differ" with regard to the period which should elapse before having recourse to craniotomy in hydrocephalic cases. Dr. Ramsbotham is of opinion that it is especially dangerous to allow a hydrocephalic head to remain for any considerable time locked in the pelvic cavity; because from its compressibility and the open state of the fontanelles, it so completely adapts itself to the shape, and moulds itself into the irregularities of the cavity, as to occasion strong, uninterrupted, and almost universal pressure, upon the lining structures, to their imminent and certain hazard,* while the fluidity of its contents adds on physical

* Ramsbotham. American Edition, p. 178.

principles to the danger of these effects. "We know of one case of the kind, in which a hydrocephalic head produced fatal laceration of the cervix uteri. In another case, where the child presented footling, the spine of the neck and part of the soft tissues, covering it, gave way under the traction employed, and the dropsical head was thus emptied and allowed to pass.* Dewees† once saw rupture of the uterus from hydrocephalus, which craniotomy, early performed, might possibly have prevented. Ramsbotham‡ relates the case of a patient who was delivered of a hydrocephalic child, who had been in labor from Sunday, when the membranes broke, to early on Friday morning, when R. first saw her; she died the same evening. Another author writes: "hydrocephalus in the child is not a common cause of protracted labor, but the diagnosis is very difficult where it is, and if the nature of the obstruction be not early ascertained, the result has generally been unfortunate. * * * * * Should the pains have continued strong for some hours, and the head have not entered the brim, the perfarater should be employed without loss of time."|| Blundell,§ wishing to guard against undue interference, condescends to be witty:—" Where the head is hydrocephalic, you may if you please, carry your hand into the uterus; you may, if you please, burst the vagina; you may, if you please, rupture the uterus, turn the child, and pull its head from its body; but have some little mercy. Give a trial of those natural efforts, which, by the wise accoucheur, are never hastily distrusted. The natural efforts failing, puncture the head, should the lever or forceps have been previously tried without success."

In breech and footling cases, these instruments are useless, and only protract a delivery which cannot be accomplished with them. Craniotomy, therefore, should be had recourse to, so soon as we are satisfied that we have made use of as much exertion as we think ourselves warranted in doing, after the head had been placed in the most favorable position.

5thly. *Size of Head.* A better idea of this may be formed by comparing it with "average size" heads of a fœtus at birth, and of a British Canadian.

| | Fœtus at Birth. | British Canadian. | Mrs. W.'s. Child. |
|---|---|---|---|
| From occipital protuberance over vertex to nasal spine, | 5½¶ | 14 | 22¾ |
| Greatest circumference, | 14 | 22¼ | 29½ |

The records of midwifery that I have been enabled to consult, afford no such instance of a hydrocephalic monster—one alone excepted:—"In 1834, Mr. T. Marsh, Coleford, Gloucester, attended Mrs. ———, in labor with her sixth child. After long delay, Mr. M. dispatched a messenger to some five miles distance for instruments, but before his return, nature—the safest of accoucheurs—had accomplished delivery. The dimensions of the child's head were as follows:—Radix nasi to protuberantia occipitalis, 26 inches; front occipital circumference, 32

* Forbes Medical Review, Vol. XII., p. 480.
† idwifery, p. 527.
‡ Lee's Midwifery, p. 42.
|| Process of Parturition, p. 272.
§ Obstetric Medicine, p. 60 and 61.
¶ In these measurements of the child's head, no allowance is made for the elongation, which usually, and sometimes to a great extent, occurs in labor.

inches; ear to ear, across vertex, 24 inches. Around chin and across vertex, 30 inches."* If these measurements be correct, we must, in order to account for unassisted delivery, suppose one of two things to have existed, either an extraordinarily capacious pelvis—such a pelvis as we sometimes "read of"—or a scalp loosely covering the fluid, or, probably, both. Excepting, therefore, Mr. Marsh's case, as an extraordinary anamoly, the largest circumference I have seen recorded is 27 inches.† Instances of hydrocephalic heads, under this in size, are numerous. Two are related by Smellie, in which large heads were expelled.‡ "I have known," says Merriman, "one hydrocephalic fœtus pass entire, whose head was *seventeen* inches; another passed alive, and lived an hour, whose head measured in circumference *twenty-two* inches; both the above labors were long and painful."§ In Perfect's case, the head extracted whole, the breech having originally presented, measured 24½ inches in circumference.|| Heads much under those related by Smellie and Merriman are occasionally met with by accoucheurs.

6th. *The Amount of Fluid.* If the size of hydrocephalic heads vary, the amount of fluid varies also.

In 1751, a Mr. H. relates a case of a fœtus where the head contained a large quantity of bloody serum.¶ Ramsbotham (père) relates two cases in which he supposed each cranium to have held many pints of fluid.* In Smellie's first case, *three* pints were collected on the cranium being punctured.† In case 20 between two and three pints were poured into the skull, through the opening by which the hydrocephalic fluid was extracted.** Mr. T. Smith, Surgeon, Great Milton, delivered a woman of a child, whose head contained *four* pints of fluid.†† In case related by Dr. Georget, *four* pints of a clear yellowish fluid were evacuated by means of a trochar previous to delivery.‡‡ A woman, pregnant for the eighth time, was delivered by Dr. Hyewier of a fœtus whose head contained *one quart* of yellowish colored serum (*L'Union Medicale.*)§§ Mr. Robertson, of Aberdeen, relates the case of a woman who died 45 hours after delivery of her eighth child, from the effects of pressure upon the organs within the pelvis caused by a hydrocephalic head which contained *four* pints of water.|||| A case is mentioned by Blanchard in which four pounds of water were evacuated from the head of a fœtus after birth.¶¶ The case related by Mr. Marsh, already alluded to, the head contained 154 ozs., or 9lbs 10 ozs. of fluid !* The amount of fluid

---

* London Medical Gazette, vol. xvii., p. 486.

† I distinctly recollect having read of a head of this circumference, but on again looking for the passage containing it I am unable to find it. This, however, makes me no less certain.

‡ Smellie's Midwifery, vol. ii., p. 14 and 210.

§ Merriman's Midwifery.

|| Ramsbotham, p. 272.

¶ Smellie's Midwifery. p. 42.

* Practical observations, part 1.

† Collection 31.

** Collection 35.

†† Lancet, 1847.

‡‡ London Medical Repository, vol. 1st.

§§ Lancet, 1847.

|||| Medical Gazette, July 13, 1840.

¶¶ Good's Study of Medicine.

* London Medical Gazette, vol. 17, p. 986.

in the case of Mrs. W.'s child, cannot be stated with anything like certainty, for we unfortunately forgot to fill the cranial cavity, a proceeding which would have saved much trouble. But, supposing the cranium to have been a paraboloid, then

The circular base being,........................29.5 inches
Altitude,.............................................. 6
The contents or solidity would be,.......... 207.75 cubic inches.

And allowing 28⅞ cubic inches to the pint, the cavity would have contained about seven and one-sixth (7 1.6) pints, wine measure. From this must be deducted the amount represented by the scalp and bones, for the measurements were external; they, being much attenuated, may be represented by 1-6th of a pint, leaving seven pints of fluid;* a quantity much greater than that in any of the cases enumerated, with the exception of Mr. Marsh's.

7th. *Amount of Brain.* I have been unable, notwitstanding diligent search, to find but two instances resembling this, in *this* particular. Sir Astley Cooper, some years ago, published a case under the somewhat attractive title of "A child without a brain."

Breschet, Surgeon *en chef* to the Foundling Hospital, in Paris, relates the case of a child who lived to the age of 12 days, whose cranium was of the ordinary size, which contained no brain whatever.† Had gestation, in Mrs W.'s case been prolonged to about a week longer, the small amount of brain which existed at the time of parturition, would have been entirely absorbed. As it was, the brain was represented by about a drachm and a half of softened, greyish matter, which might have been easily folded up, and concealed in a thimble.

*In what part of the brain, and at what period of intra uterine life was this fluid first secreted?* It should here be stated, that, during the two months preceding delivery, the patient suffered very much from heat in the right side, which compelled her to lie with cold, wet clothes applied to the part. In the abnormal position of things, the fœtal head corresponded to that tender part. Could not the inflammation within the child's cranium, attended, as it no doubt was, by increased heat, have been experienced by the mother? With a small quantity of amnial fluid, I perceive no reason for doubting that the increased heat experienced by the mother was caused by the hydrocephalic head lying in immediate contact with the abdominal parietes. And, granting this assumption, I should say, in answer to the latter question, that the fluid was secreted at, or shortly before, the period when the patient first complained of pain and heat, namely, two months prior to delivery. "Hydrocephalus—whatever its results—is originally an inflammatory affection situate in the substance of the central parts of the brain, generally terminating by *ramollissement* of those parts, combined with serous effusion,"‡ and, as an inflammatory affection, is characterized by one of the symptoms of inflammation—heat.

In answer to the former question: most probably in the ventricles, these became expanded into large elliptical cavities, and, adopting the description of the first living pathologist, "the cerebral mass around the

---

* In this calculation no allowance is made for the unevenness of the cerebral surface of the base of the skull.

† London Medical Repository, vol. 18.

‡ Abercrombie on diseases of the brain, 2nd Ed., p. 148.

ventricles, especially towards the top of the head, became attenuated. Internally and inferiorly, the serum by its pressure flattened the corpora stritaa and optic thalami, and passing into the third ventricle, it forced these bodies asunder also; the corpora quadrigemina became smoothed, the commissures stretched, and the grey commissure wasted; the pillars of the fornix were forced apart, and, with the septum, driven up against the corpus callosum."† The relative situation of things having been thus changed, the fluid still continued to increase until what remained of the brain, no longer bore any resemblance to what it should have been.

A question here naturally suggests itself: how are we to account for the fact, that the brain can have its substance absorbed; its structure completely destroyed, and yet consistent with life? While the simplest derangement of its functions, is attended with so much peril, during its scarcely less vegetative infant life.

8th. *The strabismus, talipes, and fixed flexure of the fingers and toes.* These malformed conditions—intra uterine symptoms of the congenital disease had evidently existed for a considerable period. The flexors of the fingers and toes had contracted some time prior to birth, and remaining in that condition, the palmar surfaces of the phalanges had been arrested in their development. This deformity was so great, that tenotomy could not have restored them to a straightened condition, without partial dislocation.

As obstetricians are agreed that the diagnosis of hydrocephalus, *ante partum*, is at all times difficult, might not this abnormal position of the smaller joints materially assist in forming an opinion, in arm and footling cases?

Lastly. *Recovery*—Which, notwithstanding a severe illness, was rapid and satisfactory.—*Montreal Medical Chronicle, &c.*

Montreal, July, 1856.

